# Visual place recognition from end-to-end semantic scene text features

**DOI:** 10.3389/frobt.2024.1424883

**Published:** 2024-09-16

**Authors:** Zobeir Raisi, John Zelek

**Affiliations:** ^1^ Electrical Engineering Department, Chabahar Maritime University, Chabahar, Iran; ^2^ Vision and Image Processing Laboratory, Systems Design Engineering Department, University of Waterloo, Waterloo, ON, Canada

**Keywords:** robot, localization, scene text detection, scene text recognition, scene text spotting, visual place recognition

## Abstract

We live in a visual world where text cues are abundant in urban environments. The premise for our work is for robots to capitalize on these text features for visual place recognition. A new technique is introduced that uses an end-to-end scene text detection and recognition technique to improve robot localization and mapping through Visual Place Recognition (VPR). This technique addresses several challenges such as arbitrary shaped text, illumination variation, and occlusion. The proposed model captures text strings and associated bounding boxes specifically designed for VPR tasks. The primary contribution of this work is the utilization of an end-to-end scene text spotting framework that can effectively capture irregular and occluded text in diverse environments. We conduct experimental evaluations on the Self-Collected TextPlace (SCTP) benchmark dataset, and our approach outperforms state-of-the-art methods in terms of precision and recall, which validates the effectiveness and potential of our proposed approach for VPR.

## 1 Introduction

Signage is an ubiquitous feature in our society that provides us with vital information about locations and identities through various environments like street signs, billboards, and labels. Although several classifiers are proposed to identify specific types of signage, like street signs or license plates, their applicability is limited by a lack of prior knowledge, making it difficult to extend them to general text detection and recognition in diverse environments. It is essential to overcome this limitation and make the most of the wealth of information provided by signage ubiquitously. While classical Optical Character Recognition (OCR) algorithms achieve good performance in highly constrained environments, they may fail to detect and recognize text in any place (i.e., in the wild).

Signage is a crucial element for robots to navigate and map environments. The traditional SLAM processes rely on direct or indirect features (i.e., corners of texture patches), but these features lack semantics that are inherent in text. Signage offers a global localization cue that can be particularly useful in identifying addresses or locations. Additionally, the geometric characteristics of letters and numbers on signs can facilitate as features for relative pose estimation, assuming planar or vertical alignment with the ground plane. Visual Place Recognition (VPR) (Hong et al., 2019; Li B. et al., 2023) helps robots localize with respect to previously visited places, which is essential for detecting loop closures in visual SLAM and general localization tasks. However, VPR faces several challenges, including appearance variations caused by factors like perceptual aliasing, illumination changes, viewpoint shifts, pose variations, and environmental conditions such as weather and seasons. Current techniques primarily focus on indirect feature-based approaches, such as Bag of Words (BOW) methods. The lack of semantics and feature topological relationships with BOW complicate VPR result uniqueness as many solutions may map to a single signature.

Scene Text Spotting (STS), also referred to as end-to-end scene text detection and recognition (Liu et al., 2021; Raisi et al., 2021b), is a technique that aims to locate text in images (detection) and convert them into character sequences (recognition). This approach addresses both detection and recognition tasks simultaneously. However, it inherits the challenges associated with each task, such as irregular text shapes, illumination variations, low resolution, and occlusions (Raisi et al., 2020).

Deep learning Convolutional Neural Networks (DCNNs) like VGG (Simonyan and Zisserman, 2014) and ResNet (He et al., 2015) are commonly used as feature extraction backbone for various computer vision tasks like classification (Zhang et al., 2024), object detection (Zhang et al., 2021), and scene text detection and recognition (Ren et al., 2015; Liu et al., 2016; Redmon et al., 2016; He et al., 2017). Similarly, Recurrent Neural Networks (RNNs) are used to capture sequential dependencies in text (Rumelhart et al., 1986; Hochreiter and Schmidhuber, 1997; Baek J. et al., 2019) and have shown great success on benchmark datasets. However, irregular text instances and occlusion decline the performances of these approaches. The term “irregular text” refers to text with non-standard text instances that appear in arbitrary shape, significant orientation variations, or curvature. On the other hand, when there is occlusion which is the partial or complete hiding of text characters, makes it difficult for existing methods to perform well (Shi et al., 2018; Baek Y. et al., 2019; Liu et al., 2019; Baek J. et al., 2019). Morever, CNNs have two limitations: 1) difficulty in capturing long-range dependencies (Zhu et al., 2020) and 2) challenges in adapting to input variations (Khan et al., 2022). Recent advancements in text spotting approaches (Raisi and Zelek, 2021; Kittenplon et al., 2022; Zhang et al., 2022; Raisi and Zelek, 2022) have leveraged transformers (Vaswani et al., 2017), achieving superior performance. These approaches include (Raisi and Zelek, 2021, Kittenplon et al., 2022, Zhang et al., 2022), and (Raisi and Zelek, 2022). This transformer-based method with attention mechanisms (Vaswani et al., 2017) as the main module achieved superior performance in arbitrary shape benchmark datasets (Ch’ng and Chan, 2017; Yuliang et al., 2017).

Unlike Recurrent Neural Networks (RNNs), which process information sequentially, transformers work better in analyzing the entire input sequences simultaneously. The attention mechanism, which facilitates parallel processing, enables transformers to capture complex relationships between distant elements within the input data (Vaswani et al., 2017). In scene text detection, this means effectively reasoning about the connections between characters, even those with irregular shapes or occlusions. Moreover, the attention mechanism enables transformers to selectively focus on relevant image regions, making it easier to pinpoint characters amidst cluttered backgrounds. By shifting from CNNs to transformers, we can overcome the challenges of irregular text and occlusions in scene text detection and recognition. This will pave the way for more robust and accurate text processing, an important step towards achieving reliable Visual Place Recognition (VPR) in real-world environments (Carion et al., 2020; Dosovitskiy et al., 2020; Khan et al., 2022).

Visual Place Recognition (VPR) is a computer vision task that helps the robots to recognize previously visited locations by using visual cues (Hong et al., 2019). It is designed to withstand challenges such as severe changes in illumination, blurring, and large viewpoint changes. When it comes to identifying places, text that appears in wild images (such as street signs, billboards, and shop signage) can offer valuable information that can help improve the accuracy of VPR algorithms. This is due to the highly discriminative features in such collections of text that can be used to improve place recognition based on high-level textual features.

This paper presents a new approach for Visual Place Recognition (VPR) by utilizing an extended version of the transformer-based scene text spotting model our previous work, namely, called TDRL (Raisi and Zelek, 2024), to spot low-resolution, multi-oriented, and occluded text instances that are abundant in VPR tasks. Unlike previous methods (Hong et al., 2019) that relied on separate modules for text detection and recognition, the proposed technique can directly extract text strings alongside their quadrilateral bounding box coordinates from the given input in a single end-to-end process. Moreover, the backbone of the proposed architecture benefits from a masked autoencoder (MAE) (He et al., 2021) module that empowers the whole model in capturing occluded text instances. Our main contributions are as follows.

1. We propose a scene text spotting architecture that can handle the text of arbitrary shape with quadrilateral bounding boxes coordinated alongside the word instances.
2. We provide several quantitative and qualitative ablation experiments to show the performance of the proposed model when compared with state-of-the-art (SOTA) techniques for VPR, scene text detection, and scene text spotting tasks on the SCTP (Hong et al., 2019) and ICDAR15 (Karatzas et al., 2015) datasets.
3. We conduct experiments to demonstrate that using the high-level text features obtained from the proposed scene text spotting method achieves better results than SOTA visual place recognition (VPR) techniques that rely on keypoint features.

## 2 Related work

### 2.1 Scene text spotting

Scene text spotting, also called end-to-end scene text detection and recognition, is a computer vision task that unifies the detection and recognition modules and aims to output the detected bounding box and its corresponding word strings. Several techniques have been developed by researchers for this task, which can be categorized into classical machine learning methods (Wang et al., 2011; Netzer et al., 2011; Wang et al., 2012; Neumann and Matas, 2012) and deep learning-based methods (Li et al., 2017; Liu X. et al., 2018; Lyu et al., 2018; Feng et al., 2019; Qin et al., 2019; Liao et al., 2020; Liu et al., 2020). Conventional methods (Wang et al., 2011; Netzer et al., 2011; Wang et al., 2012; Neumann and Matas, 2012) for recognizing text in a scene from end-to-end depend on manual input features to produce the final text outcomes. These methods are only effective when the background is clear, and the text is horizontal. In more challenging situations like VPR applications, these approaches may result in poor performance.

With advancement of deep learning techniques in computer vision, several scene text spotting pipelines are proposed. Early deep-learning based STS methods (Li et al., 2017; Liu X. et al., 2018) usually utilized two separate module of detection and recognition to output the final results. These methods often used Convolutional Neural Networks (CNNs) for feature extraction as detection of text instances (Simonyan and Zisserman, 2014; He et al., 2015) and after alignment they applied Recurrent Neural Networks (RNNs) (Rumelhart et al., 1986; Hochreiter and Schmidhuber, 1997) for outputting the sequence of characters. These methods were mostly designed to detect and recognize horizontal text. For instance, Li et al. (2017) proposed a pioneering text-spotting approach that utilized a shared CNN backbone for feature extraction, followed by Region-of-Interest (RoI) pooling (Ren et al., 2015) for detection and RNN-based recognition to output word instances. Later, FOTS (Fast Oriented Text Spotting) (Liu X. et al., 2018), addressed the limitations of early methods by using an anchor-free CNN-based object detection framework that improved both training and inference efficiency. Additionally, FOTS introduced a RoIRotate module to handle multi-oriented text instances.

Many architectures (Lyu et al., 2018; Feng et al., 2019; Qin et al., 2019; Liao et al., 2020; Liu et al., 2020, 2021) were developed to address the irregular text spotting challenge by adopting CNN-based segmentation networks with multiple post-processing stages to generate polygon bounding boxes for these irregular text regions. For example, Qin et al. (2019) proposed a RoI Mask module to bridge the gap between detection and recognition for capturing arbitrarily shaped text. Liu et al. (2020) introduced a Bezier curve representation for the detection stage, followed by a Bezier Align module to transform curved text instances into regular text before feeding them into an attention-based recognition network. Alternative methods (Baek et al., 2020; Raisi and Zelek, 2021) have emerged that focus on spotting individual characters and then merging them to reconstruct the final text instance with an irregular shape. These approaches offer a different perspective on tackling the challenge of irregular text in scene text spotting tasks.

Recent advancements in transformer architectures (Vaswani et al., 2017) have proven to be effective in unified architecture for scene text spotting. Several SOTA STS methods (Huang et al., 2022; Kim et al., 2022; Kittenplon et al., 2022; Zhang et al., 2022; Raisi and Zelek, 2021) incorporated transformers into their frameworks and achieved superior performance on benchmark datasets that include both regular and irregular text (Lee et al., 2020; Atienza, 2021; Raisi et al., 2021a; Fang et al., 2021; Raisi et al., 2022). For example, Kittenplon et al. (2022) used a transformer-based detector, called Deformable DETR (object DEtection with TRansformers) (Zhu et al., 2020), as the core of their framework. They developed a multi-task prediction head that can generate both word instances and bounding boxes for text of any shape. Zhang et al. (2022) also developed a transformer-based pipeline, namely, TESTR (TExt Spotting TRansformers), by leveraging the Deformable DETR as the main component of the proposed framework for arbitrary shape scene text spotting. To address challenging scenarios such as occluded text, Raisi and Zelek (2022) recently proposed an end-to-end scene text spotting framework that enhances recognition performance in adverse conditions. Their method incorporates a Masked Autoencoder (MAE) within their pipeline, which works in conjunction with a powerful Deformable DETR detector (Zhu et al., 2020) to effectively capture the arbitrary shapes of occluded text instances in natural images.

### 2.2 Text-Aided VPR

The goal of VPR is to match a query image with references from a large dataset of images taken in different locations, based on visual cues alone. Many methods have been proposed by the VPR community (Arandjelovic et al., 2016; Anoosheh et al., 2019; Li Z. et al., 2023), which have achieved superior performance on benchmarks. However, most of these VPR methods (Cummins and Newman, 2008; Milford and Wyeth, 2012; Arandjelovic et al., 2016) use low-level key point features to match the query against the reference image. Extracting high-level semantic features, such as the text object including the bounding box coordinates and the corresponding word strings, can improve the matching performance and aid navigation. Various techniques (Hong et al., 2019; Li B. et al., 2023) use a different approach for VPR by utilizing extracted 2D text instances to locate places. To achieve this, specific datasets were collected from different places that contain at least one text region to match the query and reference images in the dataset.

For instance, TextPlace (Hong et al., 2019) uses two separate modules of detection and recognition from Textboxess++ (Liao et al., 2018) to extract street and store names and billboards from real-world scenarios for place recognition. The performance of this work compared to the previous SOTA techniques that used key point features demonstrates the advantages of using text objects to handle changes in illumination and viewpoint for localization. More recently, Li et al. (2023) introduced TextSLAM, a SLAM system that integrates the Visual SLAM architecture with the text objects for the VPR application. TextSLAM incorporates semantic text features and treats them as texture-rich planar patches for precise camera pose estimation and optimization, producing more accurate and robust results.

## 3 Unified scene text spotting architecture

**FIGURE 1:**
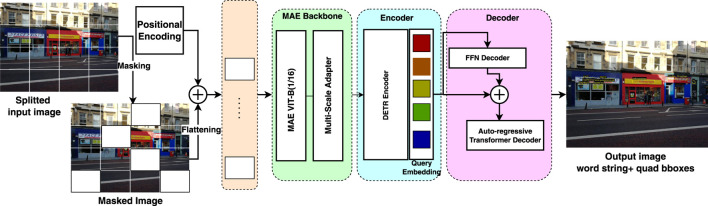
The proposed unified end-to-end scene text detection and recognition architecture for visual place recognition.

Detecting and recognizing text in a single pipeline from a given set of images is an important step for robust and efficient scene text reading. End-to-end frameworks offer significant advantages by eliminating the need for multiple processing stages, and recent advancements suggest that an end-to-end transformer-based architecture can potentially surpass the accuracy of previous end-to-end Convolutional Neural Network (CNN)-based approaches for scene text spotting tasks (Liu X. et al., 2018, 2020). In this work, we propose a single framework for unified text detection and recognition without requiring post-processing steps or ROI computations. Figure 1 provides an overview of the proposed model’s architecture. The proposed pipeline consists of three primary modules.

1. The backbone module ensures that the features are of high quality.
2. The encoder module further encodes the extracted multi-scale features from the previous stage.
3. The decoder localizes the text coordinates in terms of a quadrilateral bounding box and predicts the word strings.

The model is trained on color RGB images with quadrilateral bounding box annotations. Each annotation consists of eight coordinates $\left(x_{i},y_{i}\right)$ alongside the corresponding word string $\left(w_{s}\right)$ , structured as $g_{i}=\left[x_{1},y_{1},x_{2},y_{2},x_{3},y_{3},x_{4},y_{4},w_{s}\right]$ . The model’s output consists of quadrilateral bounding boxes with word string text instances within the given input image. These outputs are later fed into the VPR retrieval algorithm to match the query and reference frames (See Section 3.4).

The model accept an RGB image $I\in R^{H\times W\times 3}$ as input, where H and W show the height and width of I . Then, with the size of (P,P) , $N=HW/P^{2}$ are created. For example, if (H=224,W=224) and P=16 , then we create 196=14×14 patches with the size of 16×16 from the given input image. After masking a random portion of the input image and since the transformer is permutation equivalent, 1D Sinusoidal Position Encoding (1DSPE) introduced in Vaswani et al. (2017) is added to the patches.

### 3.1 VIT-based backbone

#### 3.1.1 Pre-trained MAE

After adding the 1DSPE to the patched and removing the masked patches, the remaining subset of patches are fed into the ViT Transfomer (Vit-B/16) module. This module is responsible for extracting 2D features. Vit-B/16 has repeating inner sub-blocks, each containing two essential parts: a self-attention module that analyzes relationships between different parts of the input patches and a feed-forward network that adds non-linearity. The model learns intricate relationships within the input data by stacking these modules multiple times. This allows it to capture long-range dependencies, refine features within the patches, and extract useful information about the characters. In this work, inspired by the success of Masked Autoencoders (MAE) (Li et al., 2021), We use a pre-trained Vision Transformer (ViT) architecture (Dosovitskiy et al., 2020) as the backbone for feature extraction. Initially, the input image with the size of (224×224) is divided into 196=14×14 non-overlapping patches with the size of 16×16 , and a significant portion of these patches ( ∼ 75%) are masked. One-Dimensional (1D) positional embeddings are added after the masking step, as used in the Vaswani et al. (2017).

#### 3.1.2 Multi-scale adapter

There is a crucial issue with the standard architecture of ViT that only outputs single-scale features because of its columnar structure (He et al., 2021). This problem makes the MAE ViT-B/16 backbone unsuitable for scent text detection and recognition tasks that have characters with different shapes, which require extracting multi-scale feature maps. To tackle this problem, the Multi-scale Adapter (MSA) module is added at the end of the ViT module. The multi-scale adapter module morphs the single-scale ViT features into a multi-scale Feature Pyramid Network (FPN) (Li et al., 2021; Raisi and Zelek, 2022). MSA manipulates feature maps from different encoder depths by utilizing up-sampling or down-sampling to integrate information from intermediate single-scale ViT feature with d module by using four sub-blocks that produce multi-scale features for the given resolutions input. The first two sub-blocks in MSA are up-sampled by a factor of 4 and 2, respectively. The third sub-block remains unchanged, and the fourth block is down-sampled by a factor of 2. This process creates a set of feature maps with varying resolutions (strides of 4, 8, 16, and 32 pixels relative to the input image) that encompass a wide range of spatial details.

### 3.2 DETR-based encoder

The resulting multi-scale features $\left(F^{\prime }\right)$ from MSA are then fed into a standard transformer-based encoder (E) (Zhu et al., 2020) with 6 layers to provide better semantic text features. The encoder consists of multi-heads-self-attention and Feed-forward-Network sub-blocks, enabling it to handle text instances with complex scales and resolutions. Then, the output of the encoder is set as learnable embedding queries $\left(E^{\prime }\right)$ that are later fed to the multi-task decoder. During the training phase, the encoder’s multi-head self-attention mechanism separates individual characters and word instances of the input images. It provides robust features for low-resolution and occluded text regions later used by the decoder.

### 3.3 Multi-head text spotting decoder

The decoder module is a prediction head that can output the absolute quadrilateral bounding box coordinates and the sequence of characters as word strings. The proposed multi-head prediction head eliminates the need for hand-designed components such as anchor boxes, region alignment, and non-maximum suppression used in many two-step scene text spotting methods, which decline the computational complexity and increase the inference time. As shown in Figure 1, the decoder part is different from our previous work, namely, TDRL (Raisi and Zelek, 2024). It consists of two blocks, which are described below.

#### 3.3.1 Localization decoder

The localization decoder utilizes a simple Feed-Forward-Network (FFN) to generate detection information in a format of quadrilateral bounding box coordinates of text instances from the encoded features. The outputs are then combined with learnable embedding queries of the encoder and the aggregation of themes is fed into a Transformer decoder that automatically outputs word string information.

#### 3.3.2 Recognition decoder

The recognition decoder block creates a sequence of characters using an auto-regressive transformer decoder. For each token produced, the decoder transformer uses the information of the previous token, the start location of the detected text region, and the encoded features. This process helps improve both the detection and recognition tasks during training and enables the model to be robust in capturing more challenging text instances.

### 3.4 VPR retrieval

We follow to the retrieval technique outlined in Hong et al. (2019), which utilizes the topological map generated during the mapping stage and information from scene text recognition in a new image. To calculate the similarity between the map and query image sequences, it employs a matching process that encompasses semantic (Levenshtein distance between text strings) and localization (Intersection over Union (IoU) of bounding boxes) information. The VPR retrieval process can be summarized as follows: Firstly, we extract the bounding box and word string of the input image from the output of the proposed model (Scene Text Extraction). Subsequently, we compare the extracted text information with the topological map, which contains data about previously identified scene text instances and their spatial relationships. This involves searching for matches between the extracted text and the text stored in the map (Matching with the map). In addition, the retrieval process takes into account the spatial and temporal coherence of the text to achieve more precise localization. This involves analyzing the positions of the matching texts relative to each other in both the new image and the map, which helps eliminate false positives where similar text might appear in unrelated locations (Spatial-Temporal Coherence). Based on the text matches and their spatial-temporal coherence, the location of the new image relative to the existing topological map is estimated, providing an indication of the place depicted in the new image (localization). Finally, by identifying matching text and considering their spatial relationships, the retrieval system can determine if a new image represents a previously visited location and estimate its position within the map.

## 4 Experimental results

The benchmark datasets and evaluation metrics are first defined. Subsequently, quantitative/qualitative comparisons with SOTA methods (Hong et al., 2019; Anoosheh et al., 2019; Arandjelovic et al., 2016) for the VPR task are presented. Lastly, we concluded the experimentations with an ablation study and an evaluation of computation performance.

### 4.1 Implementation details

The final model was trained on 4 NVidia GPUs RTX-3090 using 500 K cropped alphanumeric synthetic character images from the SynthText dataset (Liu et al., 2021) and 300 images from the ICDAR15 (Karatzas et al., 2015) datasets. The final proposed model that is used for evaluation is trained with a batch size of 2 per GPU. The number of object queries is set to 100 in the encoder module. The AdamW optimizer was used to optimize the model’s parameters by setting the initial learning rate to $1\times 10^{-4}$ . For augmentation, the input images are trained with the following techniques: horizontal and vertical flips, image resizing, brightness, contrast, and saturation. The evaluation and inference are done on a machine equipped with an NVIDIA RTX 3080TI GPU and 12 GB of memory. A re-implemented version of TextPlace (Hong et al., 2019) by Li B. et al. (2023) is used for comparison of qualitative results in Section 4.5. During pre-training, 75% of the input images with a resolution of 224×224 is masked out.

### 4.2 Evaluation datasets

We evaluate the performance of our proposed model using two benchmark datasets: Self-Collected TextPlace (SCTP), introduced by Hong et al. (2019), and ICDAR15 (Karatzas et al., 2015). The SCTP dataset contains images collected specifically for the VPR tasks in urban environments. It contains three map and query sequence images captured in outdoor streets and an indoor shopping mall using a mobile phone camera to simulate real-world scenarios. The SCTP evaluation dataset consists of two sets of street images for matching. The first set has 103 query images and 123 reference images, while the second set has 1097 query images and 1036 reference images. The dataset includes challenging images with high dynamic range, diverse occlusions, significant illumination variations, arbitrary-shaped text instances, and viewpoint changes, reflecting the complexities of real-world scenarios. It is worth noting that the dataset from Li B. et al. (2023) is not used in this study due to the presence of Chinese text instances which are different than the English characters used for the training of our proposed model.

The ICDAR15 (Karatzas et al., 2015) is a publicly available benchmark dataset that is designed primarily for detecting and recognizing “incidental scene text” using machine learning models. This dataset contains 1500 images for training and 500 images for evaluating end-to-end text detection and recognition algorithms. Like SCTP, these images are challenging, captured using wearable cameras both indoors and outdoors.

The Total-Text (Ch’ng and Chan, 2017) dataset is a well-known benchmark dataset specifically designed for multi-oriented and curved scene text detection and recognition. Total-Text includes 1255 images for training and 300 images for testing. In this paper, we only use the test sets of this dataset. More specifically, to evaluate the different components of the proposed pipeline in the ablation study, we use two versions of this dataset annotated at the character level as presented in Raisi and Zelek (2022). The first set contains CTT, annotated only at the character level from the original images of Total-Text. The second test set contains manually occluded characters of Total-Text, called OCTT. During the evaluation, 36 alphanumeric characters, including 10 digits + 26 capital English letters, are used.

### 4.3 Evaluation metrics

In order to fairly compare the effectiveness of our proposed model with other SOTA methods (Hong et al., 2019; Anoosheh et al., 2019; Arandjelovic et al., 2016), we use the precision-recall evaluation metric as described in Sun et al. (2019); Hong et al. (2019), which measures how well our model can accurately detect and recognize text instances for the VPR application.

To evaluate the performance of the proposed model with the current state-of-the-art (SOTA) scene text detection and end-to-end scene text detection and recognition models, we use the standard evaluation metrics introduced in Karatzas et al. (2015). These metrics include precision, recall, and H-mean (F-score) based on intersection over union (IoU). The Intersection over Union (IoU) metric is widely used in the scene text detection community to determine the accuracy of detection. To be considered accurate, a detection must have an IoU of 0.5 or greater (IOU≥0.5) . The IOU metric is defined as:

$$P=\frac{TP}{TP+FP}$$ (1)

$$R=\frac{TP}{TP+FN}$$ (2)

When evaluating scene text detection, True Positives (TP) are correctly predicted text instances, False Positives (FP) are non-text regions predicted as text regions and False Negatives (FN) are missed text regions. We can calculate the H-mean (F-score) as follows:

$$\text{H}-\text{mean}=2\times \frac{P\times R}{P+R}$$ (3)

### 4.4 SOTA VPR comparison

Table 1 presents the results of the quantitative evaluation comparing our proposed model with several state-of-the-art methods (Cummins and Newman, 2008; Milford and Wyeth, 2012; Arandjelovic et al., 2016; Anoosheh et al., 2019; Hong et al., 2019) on the SCTP dataset (Hong et al., 2019). Our model achieves the highest recall on this benchmark, which is known for containing challenging scenarios like irregular and partially occluded text instances. This superior performance in recall highlights the effectiveness of our proposed method for visual pattern recognition tasks that require robustness to such complexities.

**TABLE 1:** Quantitative comparing the proposed model with SOTA VPR techniques (Cummins and Newman, 2008; Milford and Wyeth, 2012; Arandjelovic et al., 2016; Anoosheh et al., 2019; Hong et al., 2019; Raisi and Zelek, 2024) on the SCTP (Hong et al., 2019) dataset using the Precision-Recall metric. The best results are shown in **Bold**.

Backbone	Recall				
	0.2	0.4	0.6	0.8	0.9
**Proposed**	1	1	1	**0.98**	**0.95**
TDRL	1	1	1	0.97	0.93
TextPlace	1	1	1	0.96	0.91
NetVLAD-10	1	1	1	0.95	0.93
ToDayGAN-10	0.50	0.55	0.58	0.57	0.56
FAB-MAP-10	0.79	0.69	0.67	0.65	0.63
SeqSLAM	0.30	0.24	0.18	0.13	0.13

### 4.5 VPR qualitative results

**FIGURE 2:**
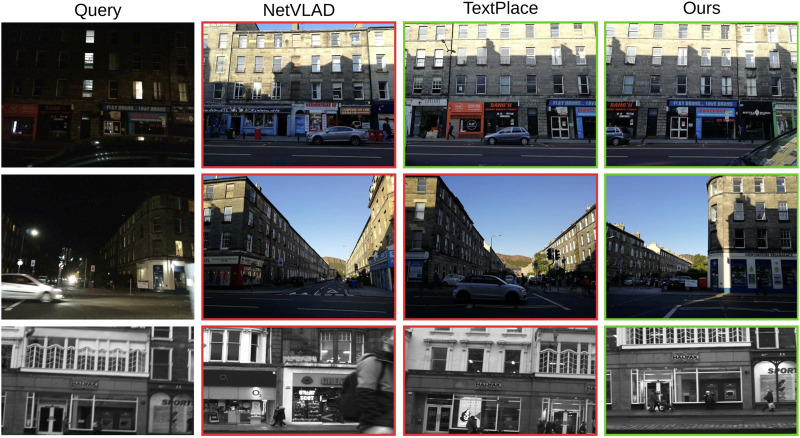
The VPR retrieval results of NetVLAD (Arandjelovic et al., 2016), TextPlace (Hong et al., 2019), and our proposed model on SCTP (Hong et al., 2019) dataset. The correct and incorrect results are shown with green and red bounding boxes. Best viewed when zoomed.

**FIGURE 3:**
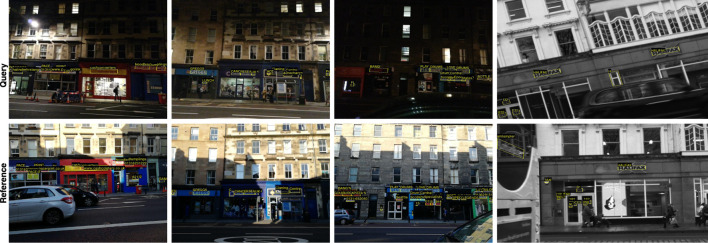
Sample query (top column)-reference (bottom column) frame pairs with text spotting results using the SCTP dataset (Hong et al., 2019). The proposed model spotted the majority of challenging cases of text instances in these frames. Best viewed when zoomed.

We evaluate the effectiveness of our text spotting model by comparing it with state-of-the-art VPR models, including NetVLAD (Arandjelovic et al., 2016) and TextPlace (Hong et al., 2019) on the SCTP dataset. Our model performs well in identifying the correct reference frame that matches the query frame, as shown in Figure 2. Notably, our model is not trained on the SCTP dataset, and it performs well without any prior knowledge of the dataset. Furthermore, we provide some qualitative results of the proposed method on challenging example images of the SCTP in Figure 3. The proposed model also shows robustness in detecting challenging text instances in both query and reference frames and is generalizable to new datasets like SCTP.

### 4.6 Ablation study

We conduct further ablation experiments and compare our proposed model with previous recent text detection and recognition techniques.

#### 4.6.1 The effect of different utilized modules in the proposed architecture

In this section, we conduct several experiments to evaluate the effect of different utilized modules in the proposed architecture, including pre-trained Masked autoencoder (MAE) backbone, training data, masking ratio, and multi-scale adapter (MSA). Table 2 shows the experimental results. To that effect, we use two subsets of alphanumeric characters of Total-Text data effectively original cropped characters of Total-Text (CTT) and occluded characters of CTT (OCTT) as described in Section 4.2 in terms of classification accuracy. We eliminated the encoder and decoder components in this experiment and only considered the backbone.

**TABLE 2:** Classification accuracy results demonstrate the effect of different modules in the proposed architecture, including the MAE backbone, masking ratio, and multi-scale adapter (MSA) module. The CTT and OCTT are original and occluded alphanumeric cropped characters of the Total-text dataset (more details Section 4.2). The best performances are shown in **bold**.

Model	Train data	Mask ratio	MSA	CTT	OCTT
ResNet-50	SynthText	–	–	86.3	83.2
ViT-B/16	SynthText	–	–	87.1	83.5
MAE	SynthText	0.65	–	89.2	86.3
MAE	SynthText	**0.75**	–	91.7	89.6
MAE	SynthText	0.85	–	90.8	88.4
MAE	SynthText	0.75	✓	92.6	90.8
**MAE**	**ImageNet + SynthText**	**0.75**	✓	**94.5**	**92.5**

We started using a CNN-based ResNet-50 backbone and trained it on the SynthText (Gupta et al., 2016) characters. It achieved 86.3% and 83.2% accuracy for the CTT and OCTT datasets, respectively. We replaced the ResNet-50 with a Transformer-based ViT-B/16 backbone slightly improved accuracy on both datasets. The accuracy was boosted when utilizing the MAE with a 0.65 masking ratio instead of the ViT-B/16 backbone on both the CTT and OCTT test sets. We then changed the masking ratio to 0.75 and 0.65 and fixed the other parameters. The model with a 0.75 masking ratio obtained the best accuracy performance. The improvement is more evident in the OCTT dataset that contains occluded characters, which confirms that masking a large portion of input image helps better in the recognition of challenging characters.

We then added the multi-scale adapter module to the model and continued with a 0.75 masking ratio; the model’s accuracy in this version also performed better than not using the MSA module. The MSA module help the model to better classify characters with different scales that are abundant in the text instances of the wild images.

Finally, we fine-tuned a pre-trained MAE backbone [trained on ImageNet (He et al., 2021)] on the Synth-Text dataset, and the model achieved the best performances on CTT (94.5%) and OCTT (92.5%) datasets. We use this fine-tuned model as the main backbone for the following experiments and training of the proposed final model.

We conducted an additional experiment to examine the impact of the backbone and encoder modules used in the proposed model. The results are shown in Table 3. The model utilizing a CNN-based (ResNet-50) architecture achieved a 64.1 F-score performance. By using an MAE-based backbone and excluding the encoder in the architecture, it outperformed the model with ResNet-50 backbone by a large margin (∼7%) . Ultimately, including the encoder in the architecture resulted in a performance boost of approximately 4% in terms of F-Score, confirming the effectiveness of leveraging the encoder in the final end-to-end text detection and recognition model.

**TABLE 3:** The effect of Transformer-based encoder in the overall architecture. The model are fine-tuned and tested on ICDAR15 datasets using the lexicon-free F-score metric. The best performance is shown in **bold**.

Backbone	Train data	Encoder	Decoder	F-score
ResNet50	ImageNet + SynthText + ICDAR15	✓	✓	64.1
MAE	ImageNet + SynthText + ICDAR15	–	✓	71.3
MAE (Proposed)	ImageNet + SynthText + ICDAR15	✓	✓	**75.4**

#### 4.6.2 SOTA text detection and recognition evaluation

We first compare the proposed model with several SOTA scene text detection and recognition approaches (Baek Y. et al., 2019; Wang et al., 2019; Zhou et al., 2017) on the benchmark ICDAR15 (Karatzas et al., 2015) dataset. The results are shown in Table 4. While the evaluation methods are trained on a combination of synthetic datasets and fine-tuned on real-world data, the proposed model achieved the highest precision (*p* = 92.1) for text detection, in addition to competitive recall and H-mean scores. The proposed model also performed well in end-to-end text detection and recognition (E2E) in terms of H-mean = 75.4. For a fair comparison, only state-of-the-art methods that have similar training image numbers to our proposed model are selected.

**TABLE 4:** The quantitative results of the proposed model in comparison among several SOTA text detection and recognition approaches on the ICDAR15 dataset. Precision, Recall, H-mean, and F-measure metrics are used to evaluate the performance of the models. The best performances are highlighted in bold.

Model	Detection			E2E	FPS
	Precision	Recall	H-mean	F-score	FPS
CRAFT Baek et al. (2019b)	88.5	84.7	86.9	–	–
PSENet Wang et al. (2019)	86.9	84.5	85.6	–	–
EAST Zhou et al. (2017)	83.3	78.3	80.7	–	–
FOTS Liu et al. (2018b)	88.8	82.0	85.3	–	–
DRGN Zhang et al. (2020)	88.5	84.6	86.5	–	–
CharNetR50 Liu et al. (2018a)	–	–	–	60.7	–
Textboxes++ Liao et al. (2018)	87.8	78.5	82.9	51.9	2.3
ABCNet-v2 Liu et al. (2021)	90.2	84.1	87.0	70.4	10
DEER Kim et al. (2022)	**93.7**	86.2	89.8	71.7	-
TESTR Zhang et al. (2022)	90.3	89.7	90.0	65.3	-
TDRL Raisi and Zelek (2024)	90.2	83.1	86.5	68.2	11.0
Proposed	92.1	**88.8**	**90.4**	**75.4**	**15.2**

#### 4.6.3 SOTA scene text spotting evaluation

**FIGURE 4:**
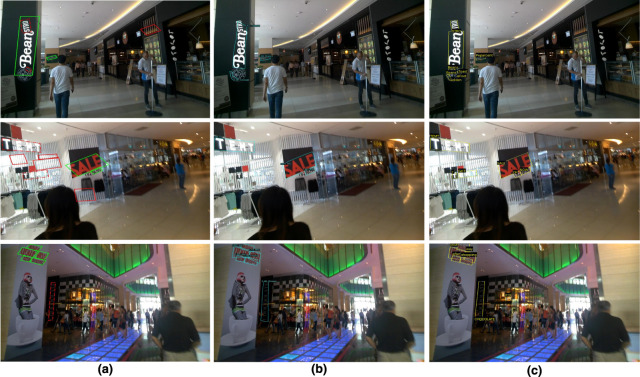
The detection and recognition results of the **(A)** textboxx++ (Liao et al., 2018) used in Hong et al. (2019), **(B)** model in Raisi and Zelek (2024), and **(C)** the proposed model. Best viewed when zoomed.

The TextPlace (Hong et al., 2019) model leveraged the Textboxes++ (Liao et al., 2018) algorithm as its main text extraction component. Therefore, we also conduct additional experiments to compare the proposed model with Textboxes++ and provide quantitative and qualitative results for text instances in the wild images using the ICDAR15 benchmark dataset. Table 4 presents a quantitative comparison between the Textboxes++, the recent TDRL technique (Raisi and Zelek, 2024), and the proposed model using the standard text detection and end-to-end spotting evaluation metrics described in section 4.3. The proposed model outperforms (Liao et al., 2018) in both detection and end-to-end spotting tasks, achieving an H-mean detection performance of 90.4% compared to 82.9%. Moreover, it surpasses Textboxes++ by approximately 27% in end-to-end F-measure performance. The proposed model also outperformed the TDRL model by a large margin, achieving a difference of ∼4% and ∼7% in both the detection and end-to-end text spotting tasks. For confirmation of these performances, we provide some qualitative STS comparisons on challenging sample images of the ICDAR15 dataset in Figure 4. As shown, the proposed model correctly detects and recognizes the text instances in the images and performs better than the TDRL and Textboxes++. We also compare the proposed model with recent state-of-the-art techniques that are equipped with contemporary ResNet + FPN backbone as in Liu et al. (2021) and transformer pipeline as in Kim et al. (2022); Zhang et al. (2022). As shown, the proposed model outperformed these methods in terms of H-mean for text detection and lexicon-free F-score for end-to-end text detection and recognition on the ICDAR15 dataset.

#### 4.6.4 Qualitative results

**FIGURE 5:**
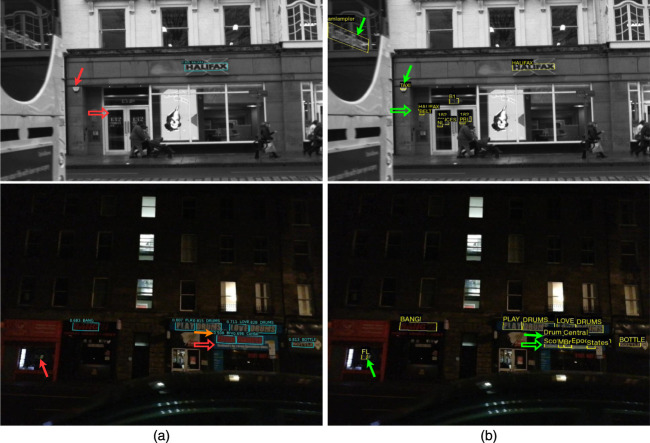
Qualitative comparison of the **(A)** proposed text spotting outputs model and **(B)** the model in Raisi and Zelek (2024). The red and green arrows illustrated the correct and missed text instances between the two models. Best viewed when zoomed.

We first provide a comparison between the qualitative results of the proposed method and the TDRL (Raisi and Zelek, 2024) on challenging example images of the SCTP in Figure 5. As shown, the proposed model effectively detects low-resolution, motion-blurred, and small text instances in images, accurately outputting corresponding word strings for the detected text regions. In contrast, the TDRL model fails to spot these challenging text instances. Successful detection of all word instances in the query frame enables the model to capture these text instances from reference frames. Outputting more detection text regions with the correct strings of the proposed model compared to the TDRL in Figure 5 and the model used in Hong et al. (2019) also affirm the good performance results in Table 1, 4.

#### 4.6.5 Semantic text versus key point features

**FIGURE 6:**
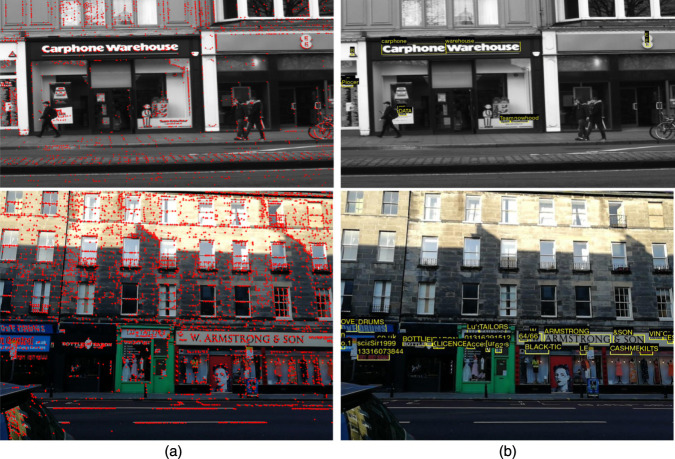
Qualitative comparison between the key point features and STS outputs. Column **(A)** shows the key points extracted from the GitHub-trained model of Zhao et al. (2022), while column **(B)** demonstrates the scene text spotting output of the proposed model. Best viewed when zoomed.

We also conducted experiments to compare qualitatively the key point features that are the output of the majority of VPR techniques and the high-level semantic text feature of the proposed model. For extracting the key points, we use the recent model in Zhao et al. (2022) that are similar but with more advanced keypoint features used in common VPR techniques. As illustrated in Figure 6, the proposed model and VPR methods differ in output features. While VPR algorithms focus on extracting key point features for place recognition tasks, the proposed model extracts semantic features with fewer numbers but with more semantic indexes.

#### 4.6.6 Inference time

Finally, we evaluated our model’s inference speed *versus* the TextPlace method (Hong et al., 2019) and used Frames Per Second (FPS) as the metric. We used an RTX 3080Ti GPU with similar memory specifications as reported in Liao et al. (2018) for TextPlace. The TDRL scene text spotting model achieved a significantly faster inference speed, reaching approximately 11 FPS compared to the 2.3 FPS reported for TextPlace (Hong et al., 2019). The proposed model shows faster inference time compared to the TDRL, achieving ∼15 FPS. We argue that by equipping the proposed model on a machine with a faster GPU, we may obtain real-time performance of 30 FPS.

## 5 Conclusions

We have developed an advanced scene text spotting model specifically designed for visual place recognition (VPR). Our model uses a pre-trained Masked Autoencoder (MAE) as a robust backbone for feature extraction and a modified multi-task transformer detector for text detection and recognition. Our experimental evaluation of the SCTP benchmark dataset shows that our proposed model surpasses the performance of state-of-the-art (SOTA) methods for VPR tasks. This highlights the effectiveness of our end-to-end approach for robust scene text detection and recognition in challenging VPR scenarios. The method can identify a revisited place chiefly based on text detected and recognized in the scene. Traditional feature-based methods can be subsequently deployed to determine the pose (i.e., translation, rotation) changes between the 2 viewing locations.

The ability to accurately detect and recognize text in the wild has the potential to revolutionize various localization and mapping tasks beyond VPR applications including augmented reality tasks. By leveraging the semantic information extracted from detected text, such methods can achieve more robust localization and mapping compared to traditional approaches that rely solely on indirect features.

## Data Availability

Publicly available datasets were analyzed in this study. This data can be found here: https://github.com/ziyanghong/dataset, https://github.com/mindspore-lab/mindocr/blob/main/docs/en/datasets/icdar2015.md.
